# Impact of *mcr-1* on the Development of High Level Colistin Resistance in *Klebsiella pneumoniae* and *Escherichia coli*

**DOI:** 10.3389/fmicb.2021.666782

**Published:** 2021-04-26

**Authors:** Xiao-Qing Zhu, Yi-Yun Liu, Renjie Wu, Haoliang Xun, Jian Sun, Jian Li, Yaoyu Feng, Jian-Hua Liu

**Affiliations:** ^1^College of Veterinary Medicine, Guangdong Provincial Key Laboratory of Veterinary Pharmaceutics Development and Safety Evaluation, Key Laboratory of Zoonosis of Ministry of Agricultural and Rural Affairs, National Risk Assessment Laboratory for Antimicrobial Resistance of Microorganisms in Animals, South China Agricultural University, Guangzhou, China; ^2^Guangdong Laboratory for Lingnan Modern Agriculture, Guangzhou, China; ^3^Biomedicine Discovery Institute and Department of Microbiology, School of Biomedical Sciences, Monash University, Clayton, VIC, Australia; ^4^College of Veterinary Medicine, Center for Emerging and Zoonotic Diseases, South China Agricultural University, Guangzhou, China

**Keywords:** *mcr-1*, *Enterobacterales*, colistin, resistance, fitness cost

## Abstract

Plasmid-mediated colistin resistance gene *mcr-1* generally confers low-level resistance. The purpose of this study was to investigate the impact of *mcr-1* on the development of high-level colistin resistance (HLCR) in *Klebsiella pneumoniae* and *Escherichia coli*. In this study, *mcr-1*-negative *K. pneumoniae* and *E. coli* strains and their corresponding *mcr-1*-positive transformants were used to generate HLCR mutants *via* multiple passages in the presence of increasing concentrations of colistin. We found that for *K. pneumoniae*, HLCR mutants with minimum inhibitory concentrations (MICs) of colistin from 64 to 1,024 mg/L were generated. Colistin MICs increased 256- to 4,096-fold for *mcr-1*-negative *K. pneumoniae* strains but only 16- to 256-fold for the *mcr-1*-harboring transformants. For *E. coli*, colistin MICs increased 4- to 64-folds, but only 2- to 16-fold for their *mcr-1*-harboring transformants. Notably, *mcr-1* improved the survival rates of both *E. coli* and *K. pneumoniae* strains when challenged with relatively high concentrations of colistin. In HLCR *K. pneumoniae* mutants, amino acid alterations predominately occurred in *crrB*, followed by *phoQ*, *crrA*, *pmrB*, *mgrB*, and *phoP*, while in *E. coli* mutants, genetic alterations were mostly occurred in *pmrB* and *phoQ*. Additionally, growth rate analyses showed that the coexistence of *mcr-1* and chromosomal mutations imposed a fitness burden on HLCR mutants of *K. pneumoniae*. In conclusion, HLCR was more likely to occur in *K. pneumoniae* strains than *E. coli* strains when exposed to colistin. The *mcr-1* gene could improve the survival rates of strains of both bacterial species but could not facilitate the evolution of high-level colistin resistance.

## Introduction

Infections caused by multidrug-resistant pathogens pose significant risks to public health worldwide (Ruhnke et al., 2014; Huang et al., 2018). Owing to a limited number of effective antimicrobial agents, colistin has been reused and is considered to be the last therapeutic agent; however, resistance to colistin has been increasingly reported (Liu et al., 2016; Srinivas and Rivard, 2017). Previously, colistin resistance was mainly caused by mutations in chromosomal genes, including *pmrAB*, *phoPQ*, *crrAB*, and the negative regulator *mgrB* (Wright et al., 2015; Poirel et al., 2017; Srinivas and Rivard, 2017), which resulted in the modification of lipid A with positively charged residues, such as phosphoethanolamine and/or 4-amino-4-deoxy-L-arabinose. However, plasmid-mediated colistin resistance genes have also emerged recently. Since, we reported the first case of plasmid-mediated colistin resistance gene (*mcr-1*) in 2015 (Liu et al., 2016), several variants of *mcr* genes (*mcr-2* to *mcr-10*) have been reported worldwide (Shi et al., 2020; Wang et al., 2020).

Similar to plasmid-mediated quinolone resistance genes, *mcr-1* generally confers low-level colistin resistance (2–8 mg/L) (Jeong et al., 2008; Jacoby et al., 2014; Liu et al., 2016). However, it is unknown whether the presence of *mcr-1* can facilitate the selection of a higher level of colistin resistance, as reported for *qnr* family genes (Jeong et al., 2008; Jacoby et al., 2014). Furthermore, the available data show that the prevalence of *mcr-1* is significantly higher in *Escherichia coli* than that in *Klebsiella pneumoniae* (Quan et al., 2017; Eiamphungporn et al., 2018). Additionally, in clinical *K. pneumoniae* isolates, colistin resistance is mostly mediated by chromosomal mutations, which usually confer higher levels of resistance (16–1,024 mg/L) than that conferred by *mcr* (Cannatelli et al., 2014; Liu et al., 2016; Jayol et al., 2017). It seems that *K. pneumoniae* is more likely to generate chromosomal mutations but is less likely to harbor *mcr-1* compared to the phenomenon observed in *E. coli*. Nevertheless, a previous study showed that after a single overnight exposure to twofold minimum inhibitory concentrations (MICs) of colistin, *mcr-1* facilitated the development of high-level colistin resistance (HLCR; MIC ≥ 32 mg/L) mutants in *E. coli* but did not affect the HLCR mutation rates in *K. pneumoniae* (Zhang et al., 2019). However, another study reported that MCR-negative strains could be induced to higher level of colistin resistance than MCR-positive strains after step-wise induction (Luo et al., 2019). Thus, the impact of *mcr-1* on the development of high level colistin resistance in *K. pneumoniae* and *E. coli*, and whether similar probability and frequency of mutations will be observed in *E. coli* and *K. pneumoniae* under selection with increasing concentrations of colistin remain unclear.

In this study, we investigated and compared the development of HLCR and chromosomal mutations among strains of *E. coli* and *K. pneumoniae*, with or without *mcr-1*, *via* multiple passages in the presence of increasing colistin concentrations.

## Materials and Methods

### Bacterial Strains and Plasmids

*Klebsiella pneumoniae* P11, HZ7H152, and YX6P94K and *E. coli* ATCC 25922, C600, and ZYTF186 were used as *mcr-1*-negative parental strains for this study. The sources and resistance phenotypes of these strains are listed in Supplementary Table 1. To construct an *mcr-1*-harboring plasmid, *mcr-1* was cloned from pHNSHP45 (GenBank accession number KP347127.1) to pHSG575 using the primer pair listed in Supplementary Table 2, which yielded pHSG575-*mcr-1* (Takeshita et al., 1987). The recombinant plasmid was transformed into *E. coli* ATCC 25922 by electroporation, whereas the original plasmid pHNSHP45 was transformed into *K. pneumoniae* P11, HZ7H152, and YX6P94K and *E. coli* C600 and ZYTF186. Transformants were selected on Luria–Bertani (LB) medium containing 2 mg/L colistin. The presence of *mcr-1* in the transformants was confirmed as per previously described methods (Liu et al., 2016).

### Whole-Genome Sequencing and Analysis

The whole genomic DNA of four strains, *K. pneumoniae* P11, HZ7H152, and YX6P94K and *E. coli* ZYTF186, was sequenced using Illumina HiSeq 2000 (Illumina, San Diego, CA, United States). Sequence reads were assembled into contigs using SOAPdenovo version 2.04. Resistance genes were explored using ResFinder^1^. The whole-genome sequences of two laboratory strains, *E. coli* C600 and ATCC 25922 (GenBank accession numbers NZ_CP031214.1 and NZ_CP009072.1, respectively), were obtained from NCBI and used as reference genomes.

### Antimicrobial Susceptibility Testing

Minimum inhibitory concentrations of colistin were determined using Mueller–Hinton (MH) broth microdilution following the recommendations of the Clinical and Laboratory Standards Institute (Matuschek et al., 2018). *E. coli* ATCC 25922 was used as a reference strain.

### Induction of Colistin Resistance by Conducting Serial Passages

Serial passaging was performed as per previously described methods (San Millan et al., 2016), with minor modifications. As shown in Supplementary Figure 1, strains were incubated on MH agar plates at 37°C to obtain single colonies. For each strain, 48 colonies were randomly selected and inoculated in separate wells of 96-well plates containing colistin-free LB broth to obtain overnight cultures. The overnight cultures were inoculated in 0.2 mL of LB broth with colistin (0.5 × MIC) in a new 96-well plate. Following a 24-h incubation at 37°C, the cultures were diluted 1:100 and transferred to a new plate containing a double concentration of colistin relative to that used on the previous day. The serial passages were performed until no surviving populations were observed. Surviving populations were defined considering growth indicated by OD_600_ (optical density at 600 nm) > 0.10 ± 0.02. The number of surviving populations was recorded for all treatments over time. For each strain, 5–10 surviving populations were selected along the concentration gradient of colistin from low to high, with priority selection of populations that survived at the highest concentration. Subsequently, the populations were separately plated on fresh LB agar to obtain single colonies, and three colonies were randomly selected from each population to determine their susceptibility to colistin and resistance mechanisms.

### Genetic Alterations in Colistin-Resistant Mutants

Chromosomal genes (*mgrB*, *pmrAB*, *phoPQ*, and *crrAB*) associated with colistin resistance were amplified by PCR and sequenced using the primers listed in Supplementary Table 2. Genetic alterations that occurred in colistin-resistant mutants were determined by comparing the resulting sequences to their corresponding parental reference genomes.

### *In vitro* Growth Rate Assay

Forty-four HLCR K. pneumoniae mutants with different mutations, including equal numbers of *mcr-1*-negative and *mcr-1*-positive derivatives, were selected incubated in fresh MH broth under shaking (180 rpm) conditions at 37°C. The overnight cultures were inoculated in fresh MH broth, and OD_600_ was measured using the Multiskan Spectrum microplate spectrophotometer (Thermo Labsystems, Franklin, MA, United States). The growth rates were determined by plotting the logarithm of OD_600_ versus time. Data analysis was performed using a non-parametric Mann–Whitney *U*-test, followed by Dunn’s multiple comparison test (Zwietering et al., 1990).

### Accession Numbers

The whole genome sequencing data of *K. pneumoniae* P11, HZ7H152, *K. variicola* YX6P94K (subtype of *K. pneumoniae*) and *E. coli* ZYTF186 have been deposited in GenBank with accession numbers JAFKAD010000000, JAFKAE010000000, JAFKAG010000000, and JAFKAF010000000, respectively.

## Results

### Development of Resistance to Colistin via Serial Passages of *Escherichia coli*

**FIGURE 1:**
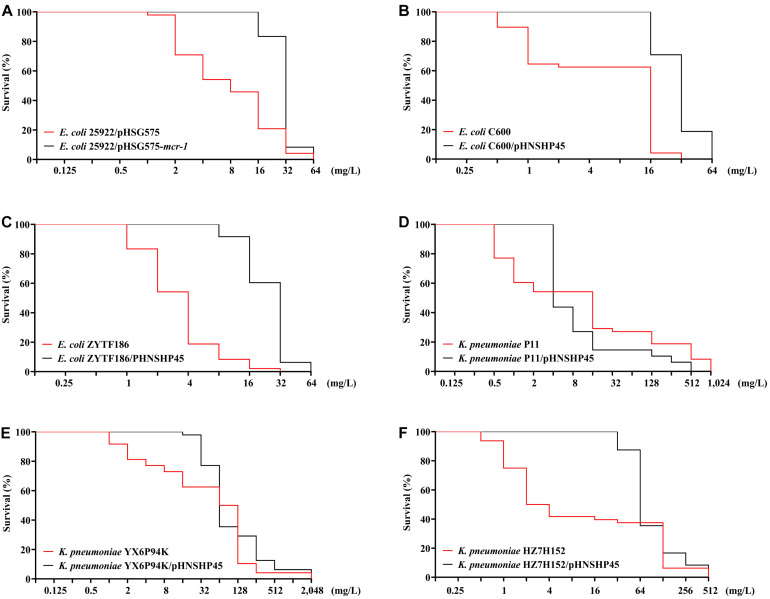
Survival curves of *E. coli* **(A–C)** and *K. pneumoniae* strains **(D–F)** and their *mcr-1*-positive transformants in the presence of increasing concentrations of colistin.

Colistin-resistant *E. coli* mutants were obtained under increasing colistin pressure. The final induction concentrations of colistin, which inhibited the growth of all *E. coli* strains, ranged from 32 to 64 mg/L. As shown in Figures 1A–C, initially, the surviving populations of the *mcr-1*-negative parental strains (ATCC 25922, C600, and ZYTF186) plummeted in response to a challenge with 2 mg/L colistin, while those of their corresponding *mcr-1*-positive derivatives did not show a marked decline until the colistin concentration reached 16 mg/L. Furthermore, when exposed to relatively high concentrations of colistin (16 and 32 mg/L), *mcr-1*-positive derivatives showed higher survival rates than those of their corresponding *mcr-1*-negative strains, demonstrating that *mcr-1* could enhance the survival rate of *E. coli* at higher colistin concentrations.

**FIGURE 2:**
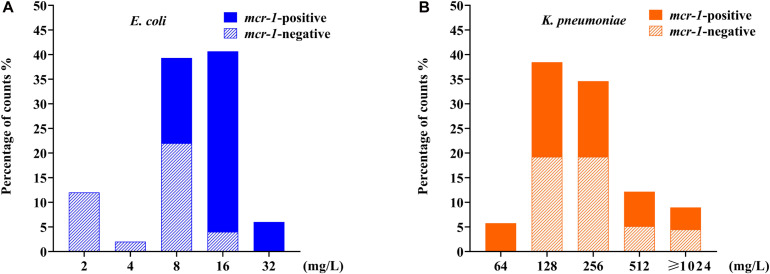
Distributions of colistin minimum inhibitory concentration (MIC) values among *E. coli* and *K. pneumoniae* mutants after induction of colistin resistance *in vitro*. **(A)** Data for 90 *mcr-1*-positive and 60 *mcr-1*-negative *E. coli* derivatives. **(B)** Data for 81 *mcr-1*-positive and 75 *mcr-1*-negative *K. pneumoniae* derivatives. *E. coli* data are indicated in blue, and *K. pneumoniae* data are indicated in orange.

After the serial colistin challenge, the highest MICs of colistin for *mcr-1*-positive *E. coli* derivatives reached 32 mg/L, while those for *mcr-1*-negative derivatives only reached 16 mg/L (Figure 2A). Colistin MIC values for all mutants ranged from 2 to 32 mg/L (*n* = 9), suggesting that it was difficult for *E. coli* to generate HLCR mutants. Compared with their basal MICs, those for the *mcr-1*-negative strains (ATCC 25922/pHSG575, C600, and ZYTF186) increased 4- to 64-fold, while the MIC values for their corresponding *mcr-1*-positive derivatives merely showed 2- to 16-fold increases, suggesting that the presence of *mcr-1* did not facilitate the generation of higher colistin resistance in *E. coli*.

### Development of Resistance to Colistin via Serial Passages of *Klebsiella pneumoniae*

Compared with *E. coli*, *K. pneumoniae* strains, with or without *mcr-1*, were more likely to generate HLCR mutations. *K. pneumoniae* strains could survive up to extremely high concentrations of colistin (256–1,024 mg/L) (Figures 1D–F). Notably, the contribution of *mcr-1* in improving the survival odds of *K. pneumoniae* was initially remarkable, however, it was weakened at higher concentrations of colistin. For example, compared with those of the parent strains of *K. pneumoniae* (YX6P94K and HZ7H152), more populations of their *mcr-1*-harboringtransformants survived when the concentrations of colistin were lower than 32 mg/L (Figures 1E,F). However, the contribution of *mcr-1* gradually diminished in the presence of >64 mg/L colistin. Furthermore, the number of P11/pHNSHP45 subsets was initially higher but decreased to fewer than that of the parent strain P11 upon exposure to more than 4 mg/L colistin (Figure 1D).

After serial passages, the colistin MIC values for *K. pneumoniae* mutants ranged from 64 to >1,024 mg/L (Figure 2B). Particularly, the colistin MICs increased 256- to 4,096-fold for *mcr-1*-negative mutants but only 16- to 256-fold for their respective *mcr-1*-harboring transformants.

### Genetic Alterations in *pmrAB* and *phoPQ* in *Escherichia coli*

Although most *E. coli* strains failed to generate HLCR, 36 derivatives that survived at relatively high concentrations of colistin (16–32 mg/L), including 18 *mcr-1*-negative and 18 *mcr-1*-positive derivatives, were selected to determine the presence of mutations in key genes related to colistin resistance (*mgrB*, *pmrAB*, and *phoPQ*). As shown in Supplementary Table 3, alterations mainly occurred in *pmrB* (21/36, 58.3%), followed by *phoQ* (15/36, 41.7%), and *pmrA* (9/36, 25.0%), most of which were associated with different amino acid substitutions, except frameshift mutations found in the *pmrB* gene (*n* = 6). No mutations were observed in *phoP* or *mgrB.*

### Genetic Alterations in *mgrB*, *pmrAB*, *phoPQ*, and *crrAB* in *Klebsiella pneumoniae*

**FIGURE 3:**
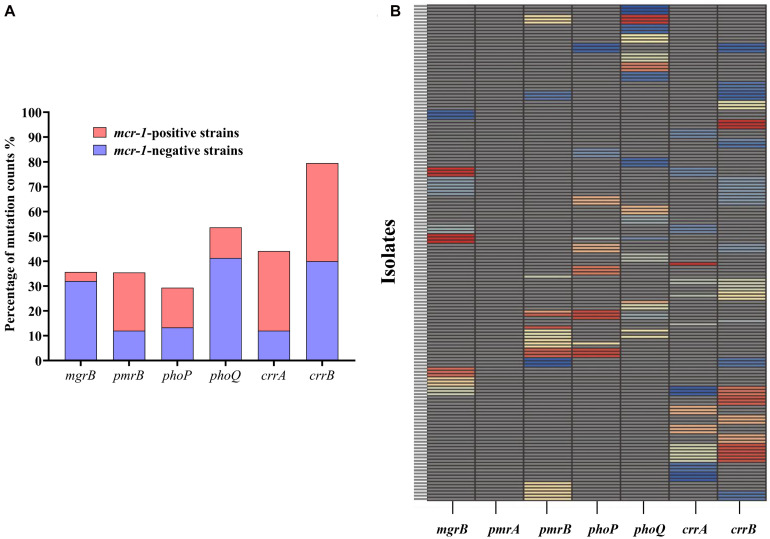
Occurrence of genetic alterations in *mgrB*, *pmrAB*, *phoPQ*, and *crrAB* in *K. pneumoniae*. **(A)** Distribution of mutations in different genes in 81 *mcr-1*-positive and 75 *mcr-1*-negative *K. pneumoniae* derivatives. **(B)** Profiles of genetic alterations in key genes related to colistin resistance, with 53 unique combinations in the 156 colistin-resistant *K. pneumoniae* derivatives. Each unique mutation in each colistin resistance gene is represented by a different color. Specific information on the mutations can be found in Supplementary Table 4.

A total of 156 HLCR *K. pneumoniae* mutants, including 75 *mcr-1*-negative and 81 *mcr-1*-positive derivatives, were examined for the presence of mutations in the *mgrB*, *pmrAB*, *phoPQ*, and *crrAB* genes. The HLCR mutants showed a remarkable multiplicity of mutations in these genes (Figure 3, Table 1, and Supplementary Table 4). Among these mutants, genetic changes predominately occurred in *crrB* (62/156, 39.7%), followed by *phoQ* (41/156, 26.3%), *crrA* (35/156, 22.4%), *pmrB* (28/156, 17.9%), *mgrB* (27/156, 17.3%), and *phoP* (23/156, 14.7%). In 55 mutants, mutations were occurred in two or three genes (Table 1). No mutations were observed in *pmrA*.

**TABLE 1:** Number of *K. pneumoniae* derivatives with genomic mutation(s) in colistin resistance genes after stepwise induction.

Genome alterations on single gene	Genetic alterations	No. of strains	No. of *mcr-1*-negative strains	No. of *mcr-1*-positive strains
*mgrB*	Insertional inactivation by ISs	9	6	3
	FMs	2	2	0
*pmrB*	FMs	5	0	5
	Amino acid substitutions	3	0	3
*phoP*	Amino acid substitutions	3	0	3
	FMs	3	0	3
*phoQ*	Amino acid substitutions	33	27	6
*crrA*	Amino acid substitutions	9	0	9
	FMs	8	0	8
*crrB*	Amino acid substitutions	21	6	15
	Amino acid substitution, FM	1	0	1
	FM	1	0	1
	Insertional inactivation by ISs, amino acid substitutions	3	0	3
Total strains with mutations in single gene		64.7% (101)	54.7% (41/75)	74.1% (60/81)
Genome alterations on two or three genes	*pmrB* and *phoQ* amino acid substitutions	5	3	2
	*phoP* and *crrB* amino acid substitutions	9	9	0
	*pmrB* and *crrB* amino acid substitutions	3	3	0
	*mgrB* FMs, *crrA* amino acid substitutions	6	6	0
	*mgrB* FMs, *crrB* amino acid substitutions	6	6	0
	*mgrB* FM, *phoPQ* amino acid substitution	1	1	0
	*crrA* FMs, *crrB* amino acid substitutions	3	0	3
	*pmrB* amino acid substitution and FM, *phoP* amino acid substitution	1	0	1
	*pmrB* and *phoPQ* amino acid substitution	1	0	1
	*phoPQ* amino acid substitution	1	0	1
	*pmrB* amino acid substitution, *phoP* FM	1	0	1
	*pmrB* and *phoP* amino acid substitutions	3	0	3
	*pmrB* FMs, *crrB* amino acid substitutions	6	3	3
	*mgrB* insertional inactivation by ISs, *crrAB* amino acid substitutions	3	3	0
	*crrA* amino acid substitutions, *crrB* FMs	6	0	6
Total strains associate with mutations in two or more genes		35.3% (55/156)	45.3% (34/75)	25.9% (21/81)

*IS, insertion sequence; FM, frameshift mutation.*

There were evident differences in mutations between the *mcr-1*-negative and *mcr-1*-positive isolates (Figure 3A, Table 1, and Supplementary Table 4). *phoQ* and *mgrB* alterations were more frequent in the *mcr-1*-negative derivatives (41.3 and 32.0%, respectively) than in the *mcr-1*-positive derivatives (12.3 and 3.7%, respectively). By contrast, the *mcr-1*-positive derivatives predominantly harbored mutations in *crrA* (26/81, 32.1%) and *pmrB* (19/81, 23.5%), both of which only occurred in 12.0% (9/75) of the *mcr-1*-negative derivatives. Moreover, of the 55 mutants with genetic change(s) in two or three genes, 34 (34/75, 45.3%) were *mcr-1*-negative derivatives and 21 (21/81, 25.9%) were *mcr-1*-positive derivatives (Table 1).

Regarding mutational patterns, alterations were mainly associated with amino acid substitutions or frameshift mutations (Table 1 and Supplementary Table 4), except for insertion sequences (ISs), which were almost exclusively associated with *mgrB* (Table 1 and Supplementary Table 4). Among 27 derivatives with alterations in *mgrB*, 18 (66.7%) were characterized by insertional inactivation of *mgrB*, which was truncated by one of the ISs, namely IS*Kpn26* (*n* = 3), IS*903B* (*n* = 6), IS*Kpn14* (*n* = 3), IS*Ecp1* (*n* = 3), or IS*1R* (*n* = 3) (Supplementary Figure 2). Additionally, *crrB* was first observed to be truncated by IS*1R* in three derivatives (Supplementary Figure 2).

### Impacts of Mutations on the Growth of HLCR *Klebsiella pneumoniae* Strains

**FIGURE 4:**
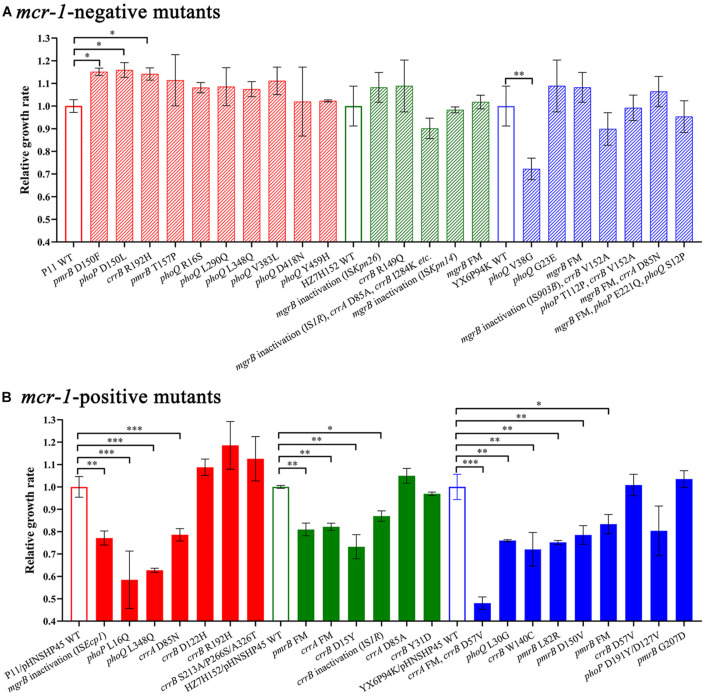
Relative growth rates of *mcr-1*-negative **(A)** and *mcr-1*-positive **(B)** *K. pneumoniae* mutants and their parental strains. The maximal growth rates are presented as relative values compared with the wild-type growth rate (set to 1.0). The relevant mutated genes of each strain are indicated below the chart. ^∗^*p* < 0.05; ^∗∗^*p* < 0.01; ^∗∗∗^*p* < 0.001. Error bars represent standard deviation values. WT, wild-type; FM, frameshift mutation.

To determine whether the genetic changes in *mgrB*, *pmrAB*, *phoPQ*, and *crrAB* could impose a fitness cost on *K. pneumoniae* strains, we determined the growth rates of HLCR mutants harboring different mutations. In the *mcr-1*-negative groups, most of the HLCR mutants showed subtle advantages in the growth rates compared with those of their wild-type parent strains (Figure 4A), except for one mutant (*phoQ* V38G), which exhibited a substantial reduction in the growth rate (*p* < 0.01), and three isolates (13.6%) with different mutations, which showed significant advantages in the growth rates (*p* < 0.05). Conversely, among the *mcr-1*-positive HLCR mutants, none exhibited remarkable growth advantages, and most mutants showed slow growth rates compared to their corresponding parent strains (Figure 4B). Additionally, 13 individual mutants (59.1%) exhibited significant biological costs for growth (*n* = 1, *p* < 0.05; *n* = 9, *p* < 0.01; *n* = 3, *p* < 0.001). Overall, most chromosomal mutations alone resulted in no burden on growth of HLCR mutants; however, the fitness cost was observed in most *mcr-1*-harboring HLCR mutants.

## Discussion

Although the presence of *mcr-1* confers low-level resistance to colistin, there is a concern regarding whether the presence of *mcr-1* can accelerate the development of HLCR. In this study, we found that *mcr-1* did not potentiate the development of HLCR in *E. coli* and *K. pneumoniae*, as the colistin MICs for *mcr-1*-negative derivatives obtained *via* serial colistin challenges were similar to those of their corresponding *mcr-1*-harboring transformants. Moreover, the fold increases in the colistin MICs were higher for *mcr-1*-negative strains than those for their *mcr-1*-harboring transformants. Although the presence of *mcr-1* could not facilitate the evolution of colistin resistance in these two species of bacteria, *mcr-1* could improve their survival rates upon exposure to relatively high concentrations of colistin (16–32 mg/L), which indicated that the presence of *mcr-1* might enhance the survival ability of bacteria under clinical colistin pressure, thereby potentially leading to treatment failure.

Previous reports have shown that the mechanisms of resistance to colistin significantly vary between clinical *K. pneumoniae* and *E. coli* isolates. In *K. pneumoniae*, colistin resistance, including HLCR, is usually caused by chromosomal mutations (Cannatelli et al., 2014; Jayol et al., 2017), whereas in *E. coli*, colistin resistance is mainly associated with the presence of *mcr-1* (Huang et al., 2017). Herein, we demonstrated that compared with *E. coli*, *K. pneumoniae* strains were more likely to develop HLCR by genetic alterations of the two-component systems (TCSs, e.g., PmrAB and PhoPQ) and their regulators (MgrB and CrrAB) when exposed to increasing concentrations of colistin *in vitro*. Previous studies have demonstrated that the TCSs play crucial roles in regulating the expression of genes for lipid A modifications in both *K. pneumoniae* and *E. coli* strains (Poirel et al., 2017; Srinivas and Rivard, 2017). This result partly explains the clinical phenomenon of HLCR being more common in *K. pneumoniae* compared to *E. coli* isolates. However, the mechanism underlying why TCSs and their regulators in *K. pneumoniae* are more likely to occur genetic alteration under the selective pressure of colistin than those in *E. coli* is unclear and needs further study.

After colistin exposure, alterations were detected in genes (*mgrB*, *pmrAB*, *phoPQ*, and *crrAB*) associated with colistin resistance. In *E. coli*, alterations mainly occurred in *pmrB*, followed by *phoQ*, and no mutations were observed in the *mgrB* or *phoP* gene. However, in HLCR *K. pneumoniae* variants, mutations were more complex and diverse and occurred in all the indicated genes, except *pmrA*. Genetic changes mainly occurred in *crrB* and *phoQ* (Figure 3A), in contrast to a previous report which stated that alterations in *mgrB* played a primary role in colistin resistance in *K. pneumoniae* (Cannatelli et al., 2014). However, consistent with previous reports (Kim et al., 2019; Yang et al., 2020), in both *E. coli* and *K. pneumoniae*, the histidine kinase genes *crrB*, *pmrB*, and *phoQ* seemed to be more common sites for mutations compared to the response regulatory genes *crrA*, *pmrA*, and *phoP*. It is possible that the sensor kinases can directly sense environmental stimuli and are therefore more likely to undergo mutations to confer protection against adverse stimuli, such as colistin pressure (Huang et al., 2020). The detected *mgrB*-disrupting ISs, IS*Kpn26*, IS*903B*, IS*Kpn14*, IS*Ecp1*, and IS*1R*, were similar to those found in other studies (Shamina et al., 2020; Yang et al., 2020). Although recent studies have shown that amino acid substitution mutations in *crrB* or IS disruptions are responsible for HLCR (Wright et al., 2015; Jayol et al., 2017; McConville et al., 2020), mutations in *crrAB* in clinical isolates have rarely been reported, possibly due to few tests performed for this newly identified two-component system and the deletion of *crrAB* in some *K. pneumoniae* strains (Wright et al., 2015). Moreover, most of the mutations observed in this study have not been previously reported; thus, further studies are warranted to elucidate the potential contributions of these novel mutations to colistin resistance.

Remarkably, there were differences in the occurrence of chromosomal mutations in *mcr-1*-positive and *mcr-1*-negative *K. pneumoniae* isolates. In most (74.1%) *mcr-1*-positive derivatives, genetic alteration(s) only occurred in single gene involved in colistin resistance, while half of the *mcr-1*-negative derivatives harbored multiple mutations, thereby implying that the coexistence of chromosomal mutations and *mcr-1* might impose a severe fitness burden on *K. pneumoniae*. To test this hypothesis, we compared the growth rates of *mcr-1*-positive and *mcr-1*-negative strains with those of their relevant HLCR mutants and found that HLCR mutants derived from *mcr-1*-negative strains exhibited almost no fitness cost, while HLCR mutants of *mcr-1*-positive strains showed an evident fitness cost. Previous studies have shown that the fitness cost attributed to chromosomal mutations or the acquisition of wild-type *mcr-1*-harboring plasmids seemed to be insignificant (Cannatelli et al., 2015; Wu et al., 2018); however, increased expression of *mcr-1* could impose a significant fitness burden on host bacteria (Yang et al., 2017; Liu et al., 2020). Our results demonstrated that the coexistence of chromosomal mutations and *mcr-1* was disadvantageous for *K. pneumoniae*, which might partly explain the lack of clinical strains with both chromosomal mutations and *mcr-1* (Nang et al., 2018).

In conclusion, we demonstrated that compared with *E. coli*, *K. pneumoniae* was more likely to develop HLCR by acquiring chromosomal mutations. Although *mcr-1* could not facilitate the selection of HLCR mutants, it could improve the survival rates of bacteria at relatively high concentrations of colistin, which might result in treatment failure.

## Data Availability Statement

The whole genome sequencing data of *K. pneumoniae* P11, HZ7H152, *Klebsiella variicola* YX6P94K (subtype of *K. pneumoniae*) and *E. coli* ZYTF186 have been deposited at GenBank with accession numbers JAFKAD000000000, JAFKAE000000000, JAFKAG000000000, and JAFKAF000000000, respectively.

## Author Contributions

J-HL designed the study. X-QZ, RW, and HX performed the experiments and collected the data. X-QZ, Y-YL, RW, and HX analyzed and interpreted the data. X-QZ and Y-YL wrote the draft of the manuscript. J-HL, Y-YL, X-QZ, JS, JL, and YF edited and revised the manuscript. All authors have read and approved the manuscript. J-HL coordinated the whole project.

## Conflict of Interest

The authors declare that the research was conducted in the absence of any commercial or financial relationships that could be construed as a potential conflict of interest.

## Footnotes

1 https://cge.cbs.dtu.dk//services/ResFinder/
