# Supplementary material for: Impact of mcr-1 on the Development of High Level Colistin Resistance in Klebsiella pneumoniae and Escherichia coli
Source: Front Microbiol. 2021 Apr 26;12:666782. doi: 10.3389/fmicb.2021.666782 (PMC8108134; doi:10.3389/fmicb.2021.666782)
Supplement: Supplementary file 1 [file Data_Sheet_1.docx]

Supplementary Materials

**
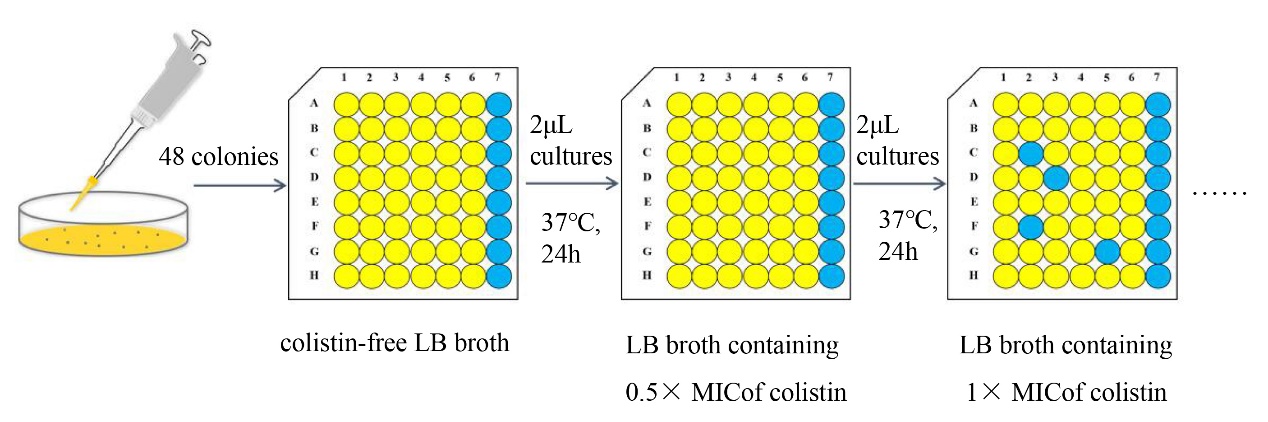
**

**Figure S1.** Flowchart of stepwise induction of colistin resistance in *K. pneumoniae* and *E.coli*. Black dots represent single colonies of a strain incubated on LB agar. Yellow wells represent surviving populations propagated in LB broth with/without colistin. Blue wells represent dead populations or a negative control.

**
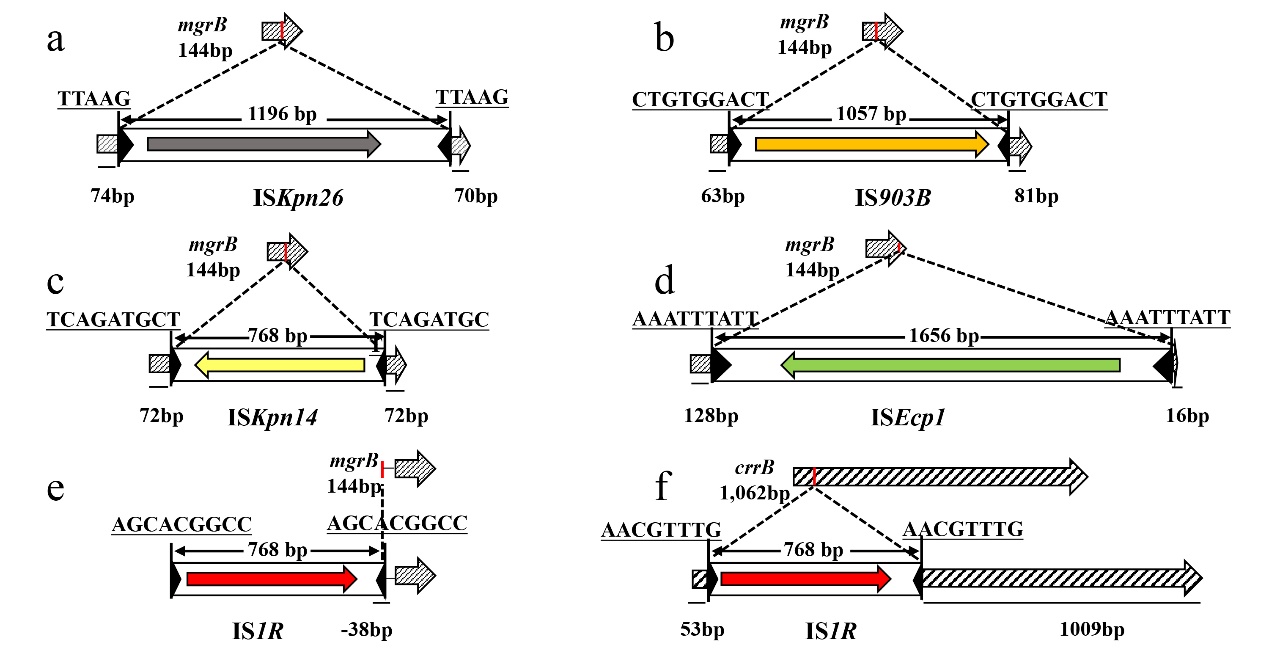
**

**Figure S2.** Schematic illustrations of insertion sequences (ISs) identified in the *mgrB* (a-e) and *crrB* (f) genes in *K. pneumoniae*. Five ISs (ISK*pn26*, IS*903B*, IS*Kpn14*, IS*Ecp1*, and IS*1R*) were identified in *mgrB*, and one IS (IS*1R*) was identified in *crrB*. Red vertical lines indicate the insertion sites, and IS event directions are represented by colored arrows, with the lengths indicated above each IS. Underlined nucleotide sequences indicate direct repeats. Black triangles flanking ISs indicate left and right inverted repeats.

**T****able S1.** Characteristics of *Klebsiella pneumoniae* (KP) and *E. coli* strains and their transformants/transconjugants used in this study

| Isolate | Species and  origin | MLST^a^ | Colistin (mg/L) | Resistance pattern^b^ | Other resistance genes |
| --- | --- | --- | --- | --- | --- |
| P11 | KP, Pork | ST11 | 0.25 | SXT, CIP, OQX, AMP, CAZ, CTX, CQ, FOX, FOS, FFC, GEN, NEO, TET, DOX | *dfrA14*, *aac*(3)-*lla, aac*(6)-*lb-cr, aph*(3’’)-*lb, aph*(6)-*ld,sul2,bla*_CTX-M15_, *bla*_OXA-1_, *bla*_SHV-187_,*bla*_-TEM1B_, *fosA*, *oqxAB*, *qnrB1*, *catB3*, *tet*(A) |
| P11/pHNSHP45 | KP, Pork | ST11 | 4 | COL, SXT, CIP, OQX, AMP, CAZ, CTX, CQ, FOX, FOS, FFC, GEN, NEO, TET, DOX | *mcr-1, dfrA14*, *aac*(3)-*lla, aac*(6)-*lb-cr ,aph*(3’’)-*lb, aph*(6)-*ld, sul2,bla*_CTX-M15_, *bla*_OXA-1_,*bla*_SHV-187_, *bla*_TEM-1B_, *fosA*, *oqxAB*, *qnrB*1, *catB3*, *tet*(A) |
| HZ7H152 | KP, Human | ST1 | 0.5 | AMP, FOS, CTX, CQ, CAZ | *bla*_CTX-M-3_*, bla*_KPC-2_*, fosA* |
| HZ7H152/pHNSHP45 | KP, Human | ST1 | 4 | COL, AMP, FOS, CTX, CQ, CAZ | *mcr-1, bla*_CTX-M-3_*_,_ blakpc-2, fosA* |
| YX6P94K | KP, Vegetable | ST2906 | 0.25 | AMP,FOS,OQX | *sul1,bla_LEN2_,aad_A2b_,fosA,oqxAB* |
| YX6P94K/pHNSHP45 | KP, Vegetable | ST2906 | 4 | COL, AMP, FOS, OQX | *mcr-1, sul1, bla*_LEN2_*, aadA2b, fosA, oqxAB* |
| ATCC25922/pHSG575 | *E. coli* | - | 0.25 | CHL | *cat* |
| ATCC25922/pHSG75-mcr-1 | *E. coli* | - | 2 | CHL, COL | *cat, mcr-1* |
| C600 | *E. coli* | - | 0.5 | STR |  |
| C600/pHNSHP45 | *E. coli* | - | 4 | STR, COL | *mcr-1* |
| ZYTF186 | *E. coli*, Human | - | 0.5 | AMP, CQ, CTX, APR, GEN, NEO, DOX, TET, CHL, FFC, FOS, SXT, CIP, OQX | *fosA, bla*_CTX-M-14_*, bla*_TEM-1b_*, dfrA, dfrA12, mdf(A), mph(A), sul1, aac(3)-IV, aadA1,aadA2,aph(3)-Ib, aph(4)-Ia, aph(6)-Id, tetA, floR, oqxAB* |
| ZYTF186/pHNSHP45 | *E. coli*, Human | - | 4 | COL, AMP, CQ, CTX, APR, GEN, NEO, DOX, TET, CHL, FFC, FOS, SXT, CIP, OQX | *mcr-1, fosA, bla*_CTX-M-14_*, bla*_TEM-1b_*, dfrA, dfrA12, mdf(A), mph**(A), sul1, aac(3)-IV, aadA1,aadA2,aph(3)-Ib, aph(4)-Ia, aph(6)-Id, tetA, floR, oqxAB* |

^a^MLST, multilocus sequence type.

^b^Antimicrobial susceptibility was determined and evaluated according to CLSI (<https://clsi.org/standards/products/microbiology/>). AMP, ampicillin; APR, apramycin; CAZ, ceftazidime; CTX, cefotaxime; CQ, cefquinome; FOX, cefoxitin; IMP, imipenem; STR, streptomycin; GEN, gentamicin; AMK, amikacin; NEO, neomycin; CHL, chloramphenicol; FFC, florfenicol; SXT, sulfamethoxazole/trimethoprim; TET, tetracycline; DOX, doxycycline, COL, colistin; CIP, ciprofloxacin; OQX, olaquindox; FOS, fosfomycin.

KP, *K .pneumoniae.*

**Table S2.** Primers used in this study

| Primer | Sequence (5’ to 3’) | | Gene | | Reference  or source |
| --- | --- | --- | --- | --- | --- |
| KP pmrA F | GACTACCAGCAGCGCTACGGCATTT | | *pmrA* | | [1] |
| pmrA R | TTCCCGTACCTCATGCTCCACATGT | |  |  |  |
| KP pmrB F | TGATGATGAAAGCCGGTAGCCCGGT | | *pmrB* | | [1] |
| pmrB R | CGCTTCTCTTATCGTCCTGCTTGCC | |  |  |  |
| KP mgrB F | GAGATACTGACACTTAAGAC | | *mgrB* | | This study |
| mgrB R | ACAACAGACCGACAAGCA | |  |  |  |
| KP phoP F | GAAGGAGTTCCATGCAGTATGCGCA | | | *phoP* | 1 |
| phoP R | TCGAAGCTAACGCTATAGCCCAC | | |  |  |
| KP phoQ F | ATGCCGAACTGCGAGAAAGCCACAC | *phoQ* | | | 1 |
| phoQ R | TCGATAAAGTCGGGCCAGTTAAGCG |  |  |  |  |
| KP crrA F | GCATGTTGTCATCAGCACTGTG | *crrA* | | | 2 |
| crrA R | GGAACCGAGTATTGCAATGG |  |  |  |  |
| KP crrB F | GGATTGAAGGGCATTCCGGA | *crrB* | | | 2 |
| crrB R | GCAGTATGTGGGATCTGTCT |  |  |  |  |
| EC pmrA F | ACGGTCTGCCTTATGCCATC | *pmrA* | | | This study |
| pmrA R | TAGCAGATAAATAGCGTCAGG |  |  |  |  |
| EC pmrB F | ATATCCATAACCTGCGCGAC | *pmrB* | | | This study |
| pmrB R | GATGATATTGACCACGAGATT |  |  |  |  |
| EC mgrB F | TTCATCACGCCTGAATTTAC | *mgrB* | | | This study |
| mgrB R | CGACTCATTCCGAAAAAGCA |  |  |  |  |
| EC phoP F | GGGTAACTCGACATGCAACT | *phoP* | | | This study |
| phoP R | TTTCACTTTACCTCCCCTCC |  |  |  |  |
| EC phoQ F | AAAGCCGCGTTTTAACACCA | *phoQ* | | | This study |
| phoQ R | ATGTACTGATGGGACGTCTG |  |  |  |  |
| pro-*mcr-1*-F | CGGAATTCCAAGATACAAATTATA  AATACTCT | *mcr-1* | | | This study |
| pro-*mcr-1*-R | ACGCGTCGACTCAGCGGATGA  ATGCGGTGCGGTC |  |  |  |  |

**Table S3.** Genomic alterations of colistin resistance genes in *E. coli*.

| Final induction concentrations  of colistin (mg/L) | Colistin induced strains | Colistin MIC  (mg/L) | Genomic alterations of colistin resistance genes |
| --- | --- | --- | --- |
| 32 | 25922/pHSG575-B5-1 | 16 | *phoQ* (A482T*,E464D) |
| 32 | 25922/pHSG575-B5-2 | 16 | *phoQ* (A482T, E464D) |
| 32 | 25922/pHSG575-B5-3 | 16 | *phoQ* (A482T, E464D) |
| 16 | 25922/pHSG575-C2-1 | 8 | *phoQ* (H18R*,V447I*) |
| 16 | 25922/pHSG575-C2-2 | 8 | *phoQ* (H18R, V447I) |
| 16 | 25922/pHSG575-C2-3 | 8 | *phoQ* (H18R,V447I) |
| 32 | 25922/pHSG575-*mcr-1*-D2-1 | 32 | *phoQ* (V435I*, H6R*) |
| 32 | 25922/pHSG575-*mcr-1*-D2-2 | 32 | *phoQ* (V435I,H6R) |
| 32 | 25922/pHSG575-*mcr-1*-D2-3 | 32 | *phoQ* (V435I,H6R) |
| 32 | 25922/pHSG575-*mcr-1*-C6-1 | 32 | *phoQ* (V435I, H6R) |
| 32 | 25922/pHSG575-*mcr-1*-C6-2 | 32 | *phoQ* (V435I, H6R) |
| 32 | 25922/pHSG575-*mcr-1*-C6-3 | 32 | *phoQ* (V435I, H6R) |
| 16 | C600-D5-1 | 8 | *pmrB* (L14P*) |
| 16 | C600-D5-2 | 8 | *pmrB* (L14P) |
| 16 | C600-D5-3 | 8 | *pmrB* (L14P) |
| 16 | C600-C2-1 | 8 | *pmrA* (S29G) |
| 16 | C600-C2-2 | 8 | *pmrA* (S29G) |
| 16 | C600-C2-3 | 8 | *pmrA* (S29G) |
| 32 | C600/pHNSHP45-C4-1 | 16 | *pmrB* (frameshift mutation at nt22) |
| 32 | C600/pHNSHP45-C4-2 | 16 | *pmrB* (frameshift mutation at nt22) |
| 32 | C600/pHNSHP45-C4-3 | 16 | *pmrB* (frameshift mutation at nt22) |
| 32 | C600/pHNSHP45-D3-1 | 16 | *pmrB* (frameshift mutation at nt22) |
| 32 | C600/pHNSHP45-D3-2 | 16 | *pmrB* (frameshift mutation at nt22) |
| 32 | C600/pHNSHP45-D3-3 | 16 | *pmrB* frameshift mutation at nt22 |
| 16 | ZYTF186-B5-1 | 8 | *pmrA* (G15E*), *pmrB* (D250E*, G283D) |
| 16 | ZYTF186-B5-2 | 8 | *pmrA* (G15E), *pmrB* (D250E, G283D) |
| 16 | ZYTF186-B5-3 | 8 | *pmrA* (G15E), *pmrB* (D250E, G283D) |
| 8 | ZYTF186-G4-1 | 8 | *pmrA* (G53E), *pmrB* (G283D), *phoQ* (A482T, E464D) |
| 8 | ZYTF186-G4-2 | 8 | *pmrA* (G53E), *pmrB* (G283D), *phoQ* (A482T, E464D) |
| 8 | ZYTF186-G4-3 | 8 | *pmrA* (G53E),*pmrB* (G283D),*phoQ* (A482T, E464D) |
| 16 | ZYTF186/pHNSHP45-B1-1 | 16 | *pmrB* (T358N*) |
| 16 | ZYTF186/pHNSHP45-B1-2 | 16 | *pmrB* (T358N) |
| 16 | ZYTF186/pHNSHP45-B1-3 | 16 | *pmrB* (T358N) |
| 32 | ZYTF186/pHNSHP45-H4-1 | 32 | *pmrB* (A159V*, Y358N*) |
| 32 | ZYTF186/pHNSHP45-H4-2 | 32 | *pmrB* (A159V, Y358N) |
| 32 | ZYTF186/pHNSHP45-H4-3 | 32 | *pmrB* (A159V, Y358N) |

**Table S4.** Genomic alterations of colistin resistance genes in HLCR *K. pneumoniae*

| Final induction concentrations of colistin (mg/L) | Colistin induced  strains | Colistin  MIC  (mg/L) | Resistance mechanism of colistin | | | | | | |
| --- | --- | --- | --- | --- | --- | --- | --- | --- | --- |
|  |  |  | *mgrB* | *pmrA* | *pmrB* | *phoP* | *phoQ* | *crrA* | *crrB* |
| 512 | P11-G2-1 | 256 | ND | ND | ND | ND | D418N* | ND | ND |
|  | P11-G2-2 | 256 | ND | ND | ND | ND | D418N | ND | ND |
|  | P11-G2-3 | 256 | ND | ND | ND | ND | D418N | ND | ND |
| 512 | P11-E1-1 | 512 | ND | ND | T157P | ND | P420L* | ND | ND |
|  | P11-E1-2 | 512 | ND | ND | T157P | ND | P420L | ND | ND |
|  | P11-E1-3 | 512 | ND | ND | T157P | ND | P420L | ND | ND |
| 512 | P11-D5-1 | 512 | ND | ND | ND | ND | L290Q* | ND | ND |
|  | P11-D5-2 | 512 | ND | ND | ND | ND | L290Q | ND | ND |
|  | P11-D5-3 | 512 | ND | ND | ND | ND | L290Q | ND | ND |
| 512 | P11-F2-1 | 256 | ND | ND | ND | ND | V383L* | ND | ND |
|  | P11-F2-2 | 256 | ND | ND | ND | ND | V383L | ND | ND |
|  | P11-F2-3 | 256 | ND | ND | ND | ND | V383L | ND | ND |
| 256 | P11-D4-1 | 128 | ND | ND | ND | D150L* | ND | ND | A145T |
|  | P11-D4-2 | 128 | ND | ND | ND | D150L | ND | ND | A145T |
|  | P11-D4-3 | 128 | ND | ND | ND | D150L | ND | ND | A145T |
| 256 | P11-H1-1 | 256 | ND | ND | ND | ND | L348Q | ND | ND |
|  | P11-H1-2 | 256 | ND | ND | ND | ND | L348Q | ND | ND |
|  | P11-H1-3 | 256 | ND | ND | ND | ND | L348Q | ND | ND |
| 256 | P11-E4-1 | 128 | ND | ND | ND | ND | Y459H* | ND | ND |
|  | P11-E4-2 | 128 | ND | ND | ND | ND | Y459H | ND | ND |
|  | P11-E4-3 | 128 | ND | ND | ND | ND | Y459H | ND | ND |
| 256 | P11-B6-1 | 128 | ND | ND | ND | ND | R16S* | ND | ND |
|  | P11-B6-2 | 128 | ND | ND | ND | ND | R16S | ND | ND. |
|  | P11-B6-3 | 128 | ND | ND | ND | ND | R16S | ND | ND |
| 256 | P11-B2-1 | 128 | ND | ND | ND | ND | ND | ND | R192H |
|  | P11-B2-2 | 128 | ND | ND | ND | ND | ND | ND | R192H |
|  | P11-B2-3 | 128 | ND | ND | ND | ND | ND | ND | R192H |
| 256 | P11-D1-1 | 128 | ND | ND | D150F* | ND | ND | ND | A145T* |
|  | P11-D1-2 | 128 | ND | ND | D150F | ND | ND | ND | A145T |
|  | P11-D1-3 | 128 | ND | ND | D150F | ND | ND | ND | A145T |
| 256 | P11/pHNSHP45-A3-1 | 256 | ND | ND | ND | ND | ND | ND | S213A, P266S, A326T |
|  | P11/pHNSHP45-A3-2 | 256 | ND | ND | ND | ND | ND | ND | S213A, P266S, A326T |
|  | P11/pHNSHP45-A3-3 | 256 | ND | ND | ND | ND | ND | ND | S213A, P266S, A326T |
| 256 | P11/pHNSHP45-C4-1 | 512 | *ISECP1*(+129bp) | ND | ND | ND | ND | ND | ND |
|  | P11/pHNSHP45-C4-2 | 512 | *ISECP1*  (+129bp) | ND | ND | ND | ND | ND | ND |
|  | P11/pHNSHP45-C4-3 | 512 | *ISECP1*(+129bp) | ND | ND | ND | ND | ND | ND |
| 256 | P11/pHNSHP45-H5-1 | 512 | ND | ND | ND | ND | ND | ND | D122H* |
|  | P11/pHNSHP45-H5-2 | 512 | ND | ND | ND | ND | ND | ND | D122H |
|  | P11/pHNSHP45-H5-3 | 512 | ND | ND | ND | ND | ND | ND | D122H |
| 128 | P11/pHNSHP45-E5-1 | 256 | ND | ND | ND | ND | ND | D85N* | ND |
|  | P11/pHNSHP45-E5-2 | 256 | ND | ND | ND | ND | ND | D85N | ND |
|  | P11/pHNSHP45-E5-3 | 256 | ND | ND | ND | ND | ND | D85N | ND |
| 128 | P11/pHNSHP45-B3-1 | 128 | ND | ND | ND | ND | ND | ND | R192H, frameshift mutation at nt542 |
|  | P11/pHNSHP45-B3-2 | 128 | ND | ND | ND | ND | ND | ND | R192H |
|  | P11/pHNSHP45-B3-3 | 128 | ND | ND | ND | ND | ND | ND | R192H |
| 128 | P11/pHNSHP45-D1-1 | 128 | ND | ND | ND | L16Q* | ND | ND | ND |
|  | P11/pHNSHP45-D1-2 | 128 | ND | ND | ND | L16Q | ND | ND | ND |
|  | P11/pHNSHP45-D1-3 | 128 | ND | ND | ND | L16Q | ND | ND | ND |
| 128 | P11/pHNSHP45-E2-1 | 128 | ND | ND | ND | ND | L348Q | ND | ND |
|  | P11/pHNSHP45-E2-2 | 128 | ND | ND | ND | ND | L348Q | ND | ND |
|  | P11/pHNSHP45-E2-3 | 128 | ND | ND | ND | ND | L348Q | ND | ND |
| 1024 | YX6P94K-A3-1 | 512 | frameshift  mutation at nt19 | ND | ND | ND | ND | D85N | ND |
|  | YX6P94K-A3-2 | 1024 | frameshift  mutation at nt19 | ND | ND | ND | ND | D85N | ND |
|  | YX6P94K-A3-3 | 512 | frameshift  mutation at nt19 | ND | ND | ND | ND | D85N | ND |
| 1024 | YX6P94K-E3-1 | 1024 | *IS903B* (+64bp) | ND | ND | ND | ND | ND | V152A |
|  | YX6P94K-E3-2 | 1024 | *IS903B* (+64bp) | ND | ND | ND | ND | ND | V152A |
|  | YX6P94K-E3-3 | 1024 | *IS903B* (+64bp) | ND | ND | ND | ND | ND | V152A |
| 512 | YX6P94K-A5-1 | >1024 | *IS903B* (+64bp) | ND | ND | ND | ND | ND | V152A |
|  | YX6P94K-A5-2 | >1024 | *IS903B* (+64bp) | ND | ND | ND | ND | ND | V152A |
|  | YX6P94K-A5-3 | >1024 | *IS903B* (+64bp) | ND | ND | ND | ND | ND | V152A |
| 256 | YX6P94K-E4-1 | 256 | ND | ND | ND | T112P* | ND | ND | V152A |
|  | YX6P94K-E4-2 | 256 | ND | ND | ND | T112P | ND | ND | V152A |
|  | YX6P94K-E4-3 | 256 | ND | ND | ND | T112P | ND | ND | V152A |
| 256 | YX6P94K-B2-1 | 256 | ND | ND | ND | ND | G23E* | ND | ND |
|  | YX6P94K-B2-2 | 256 | ND | ND | ND | ND | G23E | ND | ND |
|  | YX6P94K-B2-3 | 256 | ND | ND | ND | ND | G23E | ND | ND |
| 256 | YX6P94K-B5-1 | 256 | ND | ND | ND | ND | D127N* | ND | ND |
|  | YX6P94K-B5-2 | 256 | ND | ND | ND | ND | D127N | ND | ND |
|  | YX6P94K-B5-3 | 256 | ND | ND | ND | ND | D127N | ND | ND |
| 128 | YX6P94K-E5-1 | 128 | frameshift  mutation at nt23 | ND | ND | ND | ND | D85N | ND |
|  | YX6P94K-E5-2 | 128 | frameshift  mutation at nt23 | ND | ND | ND | ND | D85N | ND |
|  | YX6P94K-E5-3 | 128 | frameshift  mutation at nt23 | ND | ND | ND | ND | D85N | ND. |
| 128 | YX6P94K-B6-1 | 128 | frameshift  mutation at nt79 | ND | ND | ND | ND | ND | ND |
|  | YX6P94K-B6-2 | 128 | frameshift  mutation at nt79 | ND | ND | E221Q | S12P* | ND | ND |
|  | YX6P94K-B6-3 | 128 | frameshift  mutation at nt79 | ND | ND | ND | ND | ND | ND |
| 128 | YX6P94K-B4-1 | 128 | ND | ND | ND | T112P | ND | ND | V152A |
|  | YX6P94K-B4-2 | 128 | ND | ND | ND | T112P | ND | ND | V152A |
|  | YX6P94K-B4-3 | 128 | ND | ND | ND | T112P | ND | ND | V152A |
| 128 | YX6P94K-E6-1 | 128 | ND | ND | ND | ND | V38G* | ND | ND |
|  | YX6P94K-E6-2 | 128 | ND | ND | ND | ND | V38G | ND | ND |
|  | YX6P94K-E6-3 | 128 | ND | ND | ND | ND | V38G | ND | ND |
| 1024 | YX6P94K/pHNSHP45-A1-1 | 512 | ND | ND | ND | ND | ND | frameshift  mutation at nt53bp | ND |
|  | YX6P94K/pHNSHP45-A1-2 | 512 | ND | ND | ND | frameshift  mutation at nt51 | ND | ND | ND |
|  | YX6P94K/pHNSHP45-A1-3 | 512 | ND | ND | ND | frameshift  mutation at nt51 | ND | ND | ND |
| 1024 | YX6P94K/pHNSHP45-A2-1 | 1024 | ND | ND | ND | frameshift  mutation at nt505 | ND | ND | ND |
|  | YX6P94K/pHNSHP45-A2-2 | 512 | ND | ND | frameshift  mutation at nt451 | ND | ND | ND | ND |
|  | YX6P94K/pHNSHP45-A2-3 | 512 | ND | ND | ND | ND | ND | Frameshift mutation at nt53bp | D57V |
| 256 | YX6P94K/pHNSHP45-A3-1 | >1024 | ND | ND | ND | ND | ND | Frameshift mutation at nt53bp | D57V |
|  | YX6P94K/pHNSHP45-A3-2 | >1024 | ND | ND | ND | ND | ND | ND | D57V |
|  | YX6P94K/pHNSHP45-A3-3 | >1024 | ND | ND | ND | ND | ND | ND | D57V |
| 256 | YX6P94K/pHNSHP45-A4-1 | >1024 | ND | ND | ND | ND | ND | ND | W140C |
|  | YX6P94K/pHNSHP45-A4-2 | >1024 | ND | ND | ND | ND | ND | Frameshift mutation at nt53bp | W140C |
|  | YX6P94K/pHNSHP45-A4-3 | >1024 | ND | ND | ND | ND | ND | ND | W140C |
| 256 | YX6P94K/pHNSHP45-A5-1 | 256 | ND | ND | ND | ND | L30G*, L283A | ND | ND |
|  | YX6P94K/pHNSHP45-A5-2 | 256 | ND | ND | ND | ND | L30G | ND | ND |
|  | YX6P94K/pHNSHP45-A5-3 | 256 | ND | ND | ND | ND | L30G | ND | ND |
| 256 | YX6P94K/pHNSHP45-A6-1 | 256 | ND | ND | L82R, frameshift mutation at nt892 | D191Y* | ND | ND | ND |
|  | YX6P94K/pHNSHP45-A6-2 | 256 | ND | ND | L82R | D191Y | D127N | ND | ND |
|  | YX6P94K/pHNSHP45-A6-3 | 256 | ND | ND | ND | D191Y | D127N | ND | ND |
| 128 | YX6P94K/pHNSHP45-B2-1 | 128 | ND | ND | ND | ND | ND | ND | Frameshift mutation at nt23bp |
|  | YX6P94K/pHNSHP45-B2-2 | 128 | ND | ND | ND | ND | ND | Frameshift mutation at nt53bp | ND |
|  | YX6P94K/pHNSHP45-B2-3 | 128 | ND | ND | Frameshift mutation at nt15bp | ND | ND | ND | ND |
| 128 | YX6P94K/pHNSHP45-B3-1 | 128 | ND | ND | G207D* | ND | T71G*, V24G | ND | ND |
|  | YX6P94K/pHNSHP45-B3-2 | 128 | ND | ND | G207D | ND | ND | ND | ND |
|  | YX6P94K/pHNSHP45-B3-3 | 128 | ND | ND | G207D | ND | T71G, T207G | ND | ND |
| 64 | YX6P94K/pHNSHP45-B5-1 | 64 | ND | ND | D150V | ND | ND | N | ND |
|  | YX6P94K/pHNSHP45-B5-2 | 64 | ND | ND | D150V | Frameshift mutation at nt142bp | ND | ND | ND |
|  | YX6P94K/pHNSHP45-B5-3 | 64 | ND | ND | D150V | ND | ND | ND | ND |
| 64 | YX6P94K/pHNSHP45-B6-1 | 64 | ND | ND | L82R | D191Y | ND | ND | ND |
|  | YX6P94K/pHNSHP45-B6-2 | 64 | ND | ND | L82R | D191Y | ND | ND | ND |
|  | YX6P94K/pHNSHP45-B6-3 | 64 | ND | ND | L82R | D191Y* | ND | ND | ND |
| 256 | HZ7H152-H4-1 | 256 | ND | ND | Frameshift mutation at nt691 | ND | ND | ND | R192H |
|  | HZ7H152-H4-2 | 256 | ND | ND | Frameshift mutation at nt691 | ND | ND | ND | R192H |
|  | HZ7H152-H4-3 | 256 | ND | ND | Frameshift mutation at nt691 | ND | ND | ND | R192H |
| 256 | HZ7H152-B4-1 | 256 | *ISKpn26*(+75bp) | ND | ND | ND | ND | ND | ND |
|  | HZ7H152-B4-2 | 256 | *ISKpn26*(+75) | ND | ND | ND | ND | ND | ND |
|  | HZ7H152-B4-3 | 256 | *ISKpn26*(+75bp) | ND | ND | ND | ND | ND | ND |
| 256 | HZ7H152-F1-1 | 256 | *ISKpn14*(+73bp) | ND | ND | ND | ND | ND | ND |
|  | HZ7H152-F1-2 | 256 | *ISKpn14*(+73bp) | ND | ND | ND | ND | ND | ND |
|  | HZ7H152-F1-3 | 256 | *ISKpn14*(+73bp) | ND | ND | ND | ND | ND | ND |
| 256 | HZ7H152-B2-1 | 256 | *IS1* family transposase *IS1R*(-38bp) | ND | ND | ND | ND | D85A* | I284V*, Q287K, S266P |
|  | HZ7H152-B2-2 | 256 | *IS1* family transposase *IS1R*(-38bp) | ND | ND | ND | ND | D85A | I284V, Q287K  , S266P |
|  | HZ7H152-B2-3 | 256 | *IS1 family transposase IS1R*(-38bp) | ND | ND | ND | ND | D85A | I284V, Q287K, S266P |
| 128 | HZ7H152-D5-1 | 128 | ND | ND | ND | ND | ND | ND | R149Q* |
|  | HZ7H152-D5-2 | 128 | ND | ND | ND | ND | ND | ND | R149Q |
|  | HZ7H152-D5-3 | 128 | ND | ND | ND | ND | ND | ND | R149Q |
| 256 | HZ7H152/pHNSHP45-D2-1 | 256 | ND | ND | ND | ND | ND | Frameshift mutation at nt53 | ND |
|  | HZ7H152/pHNSHP45-D2-2 | 256 | ND | ND | ND | ND | ND | Frameshift mutation at nt53 | ND |
|  | HZ7H152/pHNSHP45-D2-3 | 256 | ND | ND | ND | ND | ND | Frameshift mutation at nt53 | ND |
| 256 | HZ7H152/pHNSHP45-C2-1 | 256 | ND | ND | ND | ND | ND | ND | Insertion Sequence *IS1R*(+53bp); D51N* |
|  | HZ7H152/pHNSHP45-C2-2 | 256 | ND | ND | ND | ND | ND | ND | Insertion Sequence *IS1R*(+53bp); D51N* |
|  | HZ7H152/pHNSHP45-C2-3 | 256 | ND | ND | ND | ND | ND | ND | Insertion Sequence *IS1R*(+53bp); D51N* |
| 256 | HZ7H152/pHNSHP45-C3-1 | 256 | ND | ND | ND | ND | ND | Frameshift mutation at nt53 | ND |
|  | HZ7H152/pHNSHP45-C3-2 | 256 | ND | ND | ND | ND | ND | Frameshift mutation at nt53 | ND |
|  | HZ7H152/pHNSHP45-C3-3 | 256 | ND | ND | ND | ND | ND | Frameshift mutation at nt53 | ND |
| 256 | HZ7H152/pHNSHP45-B3-1 | 256 | ND | ND | ND | ND | ND | ND | Y31D |
|  | HZ7H152/pHNSHP45-B3-2 | 256 | ND | ND | ND | ND | ND | ND | Y31D |
|  | HZ7H152/pHNSHP45-B3-3 | 256 | ND | ND | ND | ND | ND | ND | Y31D |
| 128 | HZ7H152/pHNSHP45-H4-1 | 128 | ND | ND | ND | ND | ND | D85G* | Frameshift  mutation at nt39 |
|  | HZ7H152/pHNSHP45-H4-2 | 128 | ND | ND | ND | ND | ND | D85G | Frameshift  mutation at nt39 |
|  | HZ7H152/pHNSHP45-H4-3 | 128 | ND | ND | ND | ND | ND | D85G | Frameshift  mutation at nt39 |
| 128 | HZ7H152/pHNSHP45-B5-1 | 128 | ND | ND | ND | ND | ND | D85G | Frameshift  mutation at nt39 |
|  | HZ7H152/pHNSHP45-B5-2 | 128 | ND | ND | ND | ND | ND | D85G | Frameshift  mutation at nt39 |
|  | HZ7H152/pHNSHP45-B5-3 | 128 | ND | ND | ND | ND | ND | D85G | Frameshift  mutation at nt39 |
| 128 | HZ7H152/pHNSHP45-C4-1 | 128 | ND | ND | ND | ND | ND | D15Y* | ND |
|  | HZ7H152/pHNSHP45-C4-2 | 128 | ND | ND | ND | ND | ND | D15Y | ND |
|  | HZ7H152/pHNSHP45-C4-3 | 128 | ND | ND | ND | ND | ND | D15Y | ND |
| 128 | HZ7H152/pHNSHP45-B1-1 | 64 | ND | ND | ND | ND | ND | D85A | ND |
|  | HZ7H152/pHNSHP45-B1-2 | 64 | ND | ND | ND | ND | ND | D85A | ND |
|  | HZ7H152/pHNSHP45-B1-3 | 64 | ND | ND | ND | ND | ND | D85A | ND |
| 128 | HZ7H152/pHNSHP45-G1-1 | 128 | ND | ND | Frameshift  mutation at nt691 | ND | ND | ND | ND |
|  | HZ7H152/pHNSHP45-G1-2 | 128 | ND | ND | Frameshift  mutation at nt691 | ND | ND | ND | ND |
|  | HZ7H152/pHNSHP45-G1-3 | 128 | ND | ND | Frameshift  mutation at nt691 | ND | ND | ND | ND |
| 128 | HZ7H152/pHNSHP45-F4-1 | 128 | ND | ND | Frameshift  mutation at nt691 | ND | ND | ND | R192H |
|  | HZ7H152/pHNSHP45-F4-2 | 128. | ND | ND | Frameshift  mutation at nt691 | ND | ND | ND | R192H |
|  | HZ7H152/pHNSHP45-F4-3 | 128. | ND | ND | Frameshift  mutation at nt691 | ND | ND | ND | R192H |

ND: not detected.

D: Aspartic acid; N: Asparagine; T: Threonine; P:Proline; L:Leucine; Q:Glutamine; V:Valine; A:Alanine; Y:Tyrosine; H:Histidine; R:Arginine; S:Serine; F:Phenylalanine; G:Glycine; E:Glutamic acid; W: Tryptophan; C: Cysteine; I: Isoleucine; K: Lysine.

*: Novel substitutions.

**Reference**

Kim SY, Choi HJ, Ko KS. (2014). Differential expression of two-component systems, pmrAB and phoPQ, with different growth phases of Klebsiella pneumoniae in the presence or absence of colistin. Curr Microbiol 69, 37-41.

Pishnian Z, Haeili M, Feizi A.(2019). Prevalence and molecular determinants of colistin resistance among commensal Enterobacteriaceae isolated from poultry in northwest of IraN. Gut Pathog 11, 2.
